# Supplementary material for: Neuronal identity is not static: An input-driven perspective
Source: PLoS Comput Biol. 2025 Dec 22;21(12):e1013821. doi: 10.1371/journal.pcbi.1013821 (PMC12755851; doi:10.1371/journal.pcbi.1013821)
Supplement: Attachment — Submitted filename: Response to reviewers.pdf [file pcbi.1013821.s008.pdf]

Dear Editor,

We thank the editor and reviewers for their thoughtful feedback on our manuscript. The reviewer comments have substantially strengthened our work. We have carefully addressed each concern and have made significant revisions to the manuscript. We have specifically addressed the concerns regarding the putative E/I classification and the quality of the voltage traces. We have also addressed the comments raised by the editors regarding the minimum dataset requirement by adding the extracted attributes along with the manuscript for reproducing figures (see the Data Availability Statement). The concern regarding the supplementary figures has also been resolved. Below are the specific reviewer's comments and our detailed responses to those comments, along with the specific changes made to the manuscript.

Reviewer #1

1. "At the same time, the manuscript would benefit from including more data on the types of neurons studied and discussing the relationship between the classification schemes and the intrinsic active and passive membrane properties (e.g., the role of potent voltage-gated currents, like Ih, D-type K-currents, etc.)"

   We appreciate this suggestion. Since we lacked genetic markers for true neuronal identities, we employed unsupervised clustering to reveal functional heterogeneity. However, we acknowledge that linking specific voltage-gated currents (e.g., I_h, D-type K+ currents) to our observed classification requires channel-level resolution data that was not available in this study. To address this limitation, we: (1) extracted passive membrane parameters (g_L, C_m, E_L) from GLIF model fitting (Figure 5), which provide indirect measures of membrane properties; and (2) developed the Multi-set Correlation and Factor Analysis (MCFA) framework (Figure 7) to compare which attribute sets systematically (AP, PB, AC, STA) best explain neuronal diversity under realistic input. We discuss this as a clear limitation (Limitations section, lines 1195-1205) and outline how future patch-clamp recordings, combined with dynamic clamp or computational modeling, could extend this work to provide mechanistic channel-level insights.

2. "In fact, the description of mathematical tools and numeric data (including figure legends) is a little lengthy, somewhat redundant, and might be condensed, e.g., sections 0.1.1 and 0.1.2 could be combined."

   As per the reviewer's suggestion, we have merged sections 0.1.1 (Stimulus dependence of neural classification using intracellular action potential waveforms) and 0.1.2 (Stimulus dependence of neural classification using action potential attributes) into one section (Stimulus dependence of neural classification using intracellular action potential waveforms and attributes). Also, significantly reduced the size of figure captions to improve readability.

3. “I would also welcome the presentation of additional electrophysiological data, e.g., families of voltage traces recorded from different types of barrel cortex neurons. Such traces can be informative in detecting peculiar features of the membrane potential that are associated with specific voltage-dependent currents.”

   Following the reviewer's suggestion, we added Supplementary Figure S7, which presents representative voltage traces from neurons in each FN cluster alongside their corresponding step-and-hold (SH) responses to 400 pA current injection. These traces illustrate how neurons with distinct clustering patterns generate qualitatively different firing behaviors under each stimulus type, and highlight voltage-dependent features that distinguish clusters.

4. “Figure legends are extensive and should be condensed to aid the readability of the manuscript.”

   As per the reviewer’s suggestion, we have condensed all figure legends to improve readability. Representative example: Figure 1 legend was reduced from 200 words to 140 words, focusing on what is shown rather than methodological details, which now reside in the Methods section. Similar condensing was applied to Figures 2-7.

5. “According to the Step and Hold protocol, the neurons were stimulated 10 times, using 40 to 400 pA levels. Did the authors use fixed, depolarizing current steps (no incrementing)? Static current protocols, while certainly not physiologically realistic inputs, can still uncover important biophysical parameters of the stimulated cells, especially when many current levels are applied (e.g., from -200 up to +200 pA). Biophysical features such as membrane resistance, - time constant, voltage sag, inward or outward rectification index, etc., can be readily extracted from the voltage traces recorded in such experiments. The extraction of biophysical features using a GLIF model (line 176) might provide less reliable estimates for the passive membrane properties than the analysis of current step data.”

   We thank the reviewer for this important technical question. To clarify: (1) The step-and-hold protocol used fixed, depolarizing current steps without increments (40-400 pA). We clarified this further in the methods section (see line 91). (2) Regarding the extraction of passive membrane parameters (g_L, C_m, etc.), we acknowledge the reviewer's valid concern that GLIF model fitting may provide less reliable estimates of passive properties than direct extraction from multi-level current step recordings. We have added this point in the discussion under the limitations section (see lines 1033-1034). However, our analysis strategy differs: the SH protocol was used specifically to compare clustering results across input conditions (primary result in Figures 1-2), while passive biophysical parameters were extracted from FN recordings using GLIF fitting. The rationale is that passive properties are theoretically input-invariant, and extracting them from physiologically realistic FN input ensures compatibility with the rest of our functional characterization under a physiologically realistic input. It is also important to mention that we simply didn’t have an equivalent SH

recording for every FN recording that is used in biophysical feature extraction.

6. “The analysis of the electrophysiological data focuses on action potential features and temporal parameters of firing (e.g., ISI-associated parameters). Clearly, this is justified, as the process of action potential generation in response to excitatory inputs represents the main function of neurons. At the same time, AP features and intrinsic excitability depend on a multitude of subthreshold voltage-gated currents, too. The impact of such currents is also better estimated from experiments when multiple levels of injected current are used.”

   We appreciate this point by the reviewer. It is correct that multi-level current injections provide more robust estimates of subthreshold currents and passive membrane properties. However, our study prioritizes action potential-based properties for comparing functional diversity across input conditions, which is the primary focus of Figures 1-2 and Section 1. For the passive biophysical analysis (Figure 5), we extracted parameters via GLIF model fitting from FN recordings. While this approach is less optimal than multi-level step protocols for passive property characterization, it provides parameters compatible with our FN-based attribute sets. Moreover, we didn’t have the corresponding SH recordings for all the neurons we studied in Section 2 in Results (Functional classification of neurons stimulated by the FN protocol based on different feature sets); therefore, GLIF-based passive feature extraction was the possible option. We have addressed this limitation in the discussion section, under limitations (see lines 1033-1034).

7. “Fig. 1d: It would be useful to show the membrane potential change of the stimulated neuron during the current step, including the transients, i.e,. the onset and termination phases. Fig. 1c: The top trace is a little weird; very sharp changes in Vm are seen, which can indicate some issues with electrode access resistance or capacitance. The two traces (red and blue) show very different neuronal behavior, but the underlying inputs seem to be different, too. Were the waveforms of the 360-s frozen noise stimuli identical for the different neurons?”

   Thank you for these important technical questions. (1) Regarding Figure 1c-d: We have revised these panels to include full transient responses showing onset and termination phases for both step-and-hold and frozen noise stimuli. The sharp membrane potential changes visible in the original Figure 1c reflect genuine physiological responses rather than recording artifacts, which are due to the shape of the FN input itself. (2) Recording quality control: All the specifications of recording are provided in the Methods section (see slice electrophysiology subsection). The recordings underwent a rigorous quality check. All recordings met strict quality criteria: series resistance was between 5-15 MΩ, and membrane potential stability ± 7 mV during the recording session. We verified capacitance compensation settings and monitored electrode access resistance

continuously. Data were band-pass filtered at 0.1–3000 Hz before storage for offline analysis. Post-hoc quality checks were also applied, and the recordings containing high-frequency noise and observed distortions were eliminated from the analysis. Only recordings meeting these criteria were included in the analysis. (3) Frozen noise inputs: The 360-s FN stimulus waveforms were identical across all neurons; the snippets shown in Figure 1.c are from different time instances since the authors wanted to include snippets consisting of spikes. We note that neurons were first observed under a microscope and then stimulated with an initial probe step current. Based on the shape of the soma, the firing pattern observed, neurons were stimulated either with a Frozen noise with a large time constant (250 ms) when they showed a triangular-shaped soma along a slow spiking response (<50 Hz), or otherwise with an input with a small time constant (50 ms). This explains the apparent variation in input waveforms shown in different figure panels. This is mentioned more explicitly in the methods section (see Frozen Noise subsection under Methods, lines 105-110).

- "Observed temporal parameters of action potentials, such as their amplitude and slope, depend on the electrode access resistance and filter parameters used during the recordings. Did the authors check the quality of the recordings and accept only the ones that meet the criteria for accurate feature extraction?"

  As stated in response to the previous comment, the recordings underwent a rigorous quality check. All recordings met strict quality criteria: series resistance was between 5–15 MΩ, and membrane potential stability was ± 7 mV during the recording session. We verified capacitance compensation settings and monitored electrode access resistance continuously. Data were band-pass filtered at 0.1–3000 Hz before storage for offline analysis. Post-hoc quality checks were also applied, and the recordings containing high-frequency noise and observed distortions were eliminated from the analysis. Only recordings meeting these criteria were included in the analysis.

- "Line 154: Inter-spike intervals of a neuron are primarily influenced by the inputs the neuron receives, so I consider ISIs as a kind of vague measure of the neuron's intrinsic properties. ISIs are strongly determined by the prior history of membrane potential and the instantaneous levels of excitation and inhibition (the same is true for the spike threshold). It is certainly not easy to decide whether parameters of APs can be (should be) averaged and measures of descriptive statistics can be effectively applied to quantify the complex process of neuronal input-output transformation. One can claim that averaging AP parameters results in a loss of information that might impact the classification. It might be a good idea to treat each action potential as a separate entity and construct higher-dimensional feature vectors from its parameters, like voltage threshold, amplitude, half-width, etc."

  We appreciate this thoughtful critique about information loss through averaging. The reviewer raises a valid concern that ISIs and spike thresholds are strongly input-dependent, making simple averaging potentially result in information loss. However, we note that treating each action potential as a separate entity creates

unequal feature dimensionality across neurons (spike counts vary in orders of magnitude between neuron types), making statistical clustering challenging. Our approach employs descriptive statistics (mean, std, min, and max values) to capture the distribution of AP parameters within each stimulus condition, which partially preserves information about heterogeneity; and this averaging approach is standard in electrophysiology literature, for example:

- The Allen Cell Types Database explicitly averages AP features across all action potentials in responses for cell type characterization (Gouwens et al., 2018; Teeter et al., 2018).
- Brain-wide neuronal classification studies average AP parameters from rheobase responses for cell type identification (Tripathy et al., 2015).
- Recent studies combining electrophysiology with molecular identity explicitly state that they average multiple AP measurements when multiple values are extracted (Roussel et al., 2023).
- The Petilla Interneuron Nomenclature consensus framework, which established standardized terminology for interneuron classification, implicitly supports using averaged descriptive statistics for electrophysiological features (Ascoli et al., 2008).

Moreover, there are measures such as firing rate and AP count that are given per trial. Taking a vector of thresholds and amplitudes will overpower such trial-level variables due to the curse of dimensionality. That said, we acknowledge this limitation and have included it in Section 0.6 (Limitations).

Reviewer #2:

1. “The main issue I have with the results as they are currently presented concerns the distinction between (putative) excitatory and inhibitory neurons. While the criterion used by the authors (broad spikes and low firing rates for excitatory neurons vs. narrow spikes and high firing rates for inhibitory neurons) is the classically accepted one, I am afraid that this classification, without any type of information about the genetic identity of the cells under analysis, is too prone to mistakes in attribution to be of practical use. For instance, there are several interneuron classes (e.g., somatostatin-positive neurons like Martinotti cells) that don’t fall neatly in either class (see, for instance, clusters 2 and 8 in Fig. 3b,c). Therefore, without genetic access to these cells, there is no way of knowing for sure whether such neurons are in the dataset considered by the authors and, if that is the case, in which cluster they have been placed. In my opinion, this greatly reduces the usefulness of the results presented in Figs. 4-7, since one is never 100% that what the authors call “excitatory” or “inhibitory” cells indeed belong to that specific class.”

   We appreciate this critical concern about cell type classification without genetic markers. We agree that electrophysiological classification is prone to misattribution, particularly for interneuron subtypes like somatostatin-positive (SST+) Martinotti cells. This limitation is

why we consistently use the term 'putative' E/I classifications throughout. Our primary objective was not to provide definitive cell type identification, but to demonstrate that putative E/I populations exhibit substantial functional diversity under different stimulation conditions, and to use the MCFA framework for identifying which attribute sets best explain this diversity. Regarding clusters 2 and 8 in Figure 3b-c (which the reviewer noted don't fit canonical E/I criteria): we acknowledge that these clusters may indeed contain SST+ or other interneuron subtypes that don't clearly segregate by spike width/firing rate criteria. This ambiguity illustrates precisely why genetic validation is essential. Our framework's utility is not diminished by this limitation, but rather, the methodology demonstrates that functional heterogeneity exists independent of definitive cell type assignment. Future experiments combining our FN stimulation protocol with transgenic markers (Pv-Cre for basket cells, SST-Cre for Martinotti cells) or post-hoc transcriptomics would resolve these ambiguities and strengthen the cell type-phenotype relationship. We have strengthened the discussion, clarifying this limitation (see lines 1022-1030).

2. “Also, I think the authors should justify better why they used features extracted from the FN traces to distinguish between putative excitatory and inhibitory cells, rather than from the SH traces: this is because firing rates in the range 5 to 10 spikes/s can be hardly considered “high frequencies” when parvalbumin-positive basket cells can fire up to 100 spikes/s in response to a DC step of current.”

   We appreciate this important question. The reviewer correctly points out that basket cells (Pv+) can fire at 100+ spikes/s, making our observed 5-10 spikes/s range seem modest. We used FN-derived features for E/I classification for the following reasons:

   1. Different stimulus conditions produce different absolute firing rates. Under current-clamp somatic current injection (as in our experiments), even fast-spiking basket cells fire at lower rates than they would under natural synaptic conditions or voltage-clamp protocols that maximize firing. The critical point is not absolute firing rate, but the relative distinction between populations: FN recordings consistently separated neurons into two groups with distinct firing characteristics (~5-10 spikes/s for the putatively inhibitory group vs. ~1-3 spikes/s for the putatively excitatory group), which aligns with classical broad/narrow spike-width distinctions (Figure 3b).
   2. FN stimulation more closely approximates in vivo input dynamics than step injection, making it a more appropriate reference stimulus for functional classification.
   3. The SH-FN overlapping dataset contained 185 neurons, while FN recordings included 312 neurons, providing improved statistical resolution for identifying robust functional subtypes.

   Nonetheless, we acknowledge that this classification remains 'putative' without genetic markers. We have clarified this language throughout and discussed it explicitly in Section 0.6 (Limitations: lines 1022-1030) and in Section “Functional classification of neurons

stimulated by the FN protocol based on different feature sets" (lines 663-664). We recognize that future studies combining FN stimulation with genetic markers (Pv-Cre, SST-Cre) would validate and refine these boundaries.

3. "The description of the frozen noise protocol is not clear."

   We appreciate this feedback. We have expanded the Frozen Noise Protocol section in Methods to include: (1) detailed mathematical description of the hidden state Markov process generating the stimulus waveform, (2) specification of the exponential kernels and time constants used, (3) the exact weight matrix W connecting hidden states to somatic current injection, and (4) concrete parameter values used in all simulations. We also clarify that FN waveforms were generated identically for all neurons, with variations in neuronal response reflecting genuine heterogeneity. We believe these additions substantially improve clarity (see lines 94-113).

4. "The authors claim that the FN protocol is more biologically realistic than the step and hold one. While I agree with this claim, the voltage traces shown in Fig. 1c, particularly in the top panel, do not look very realistic either: the jumps in the membrane potential induced by the stimulation appear to be excessively large, especially compared to what one would expect to see in vivo."

   We appreciate this observation. The sharp voltage transients in Figure 1c result from our use of exponential filters in the FN stimulus generation and the absence of gradual rise times in the underlying hidden state dynamics. You are correct that these jumps represent a limitation of the current FN protocol; they are sharper than what neurons experience in vivo, where synaptic integration typically occurs across dendrites with slower dynamics.

   However, we note that: (1) the FN stimulus remains substantially more physiologically realistic than step-and-hold current injection, which is completely absent in vivo; (2) despite the stimulus's limitations, it successfully reveals input-dependent functional heterogeneity that is not revealed by SH input alone (Figures 1-2).

   For full biological realism, future work could incorporate longer synaptic time constants and/or a dynamic clamp input protocol. Nonetheless, the current FN protocol provides valuable insights into functional heterogeneity. We have clarified these limitations in Section 0.6 and added a discussion of how more sophisticated stimulation protocols could further enhance biological relevance.

5. "Since the cluster structure in Fig. 1a-b looks somewhat similar in the FN and SH conditions, I think it would be more clear for the reader if the clusters were numbered and colored in a way that makes interpretation more straightforward: for example, clusters 2 and 6 in the FN condition should have the same numbers and colors as clusters 3 and 4 in the SH condition, respectively. While I realize that this is not possible for all clusters (e.g., cluster 5 in the SH condition does not seem to have a clear

equivalent in the FN condition), I think the aim of this reordering should be to maximize the values of cosine similarity on the diagonal of the heat map in Fig. 1f.”

We appreciate this thoughtful suggestion for improving figure clarity. However, after careful consideration, we concluded that reordering is not appropriate for this analysis. The cosine similarity heatmap (Figure 1f) reveals many-to-many mappings between FN and SH clusters rather than a clean one-to-one correspondence. This reflects our central finding: functional neuronal classification is fundamentally input-dependent, not merely a relabeling of identical population structure.

Forcing diagonal alignment through reordering would obscure this critical insight. Additionally, since our analysis incorporates four attribute sets - including abstract features (AC, STA) that cannot be visualized in UMAP space - reordering based solely on waveform similarity would misrepresent the multidimensional heterogeneity. The off-diagonal elements in the heatmap quantify the degree of cluster reorganization across stimulus types, making them informationally critical rather than noise. We have clarified the heatmap interpretation in the Figure 1 legend to help readers understand how to read this visualization.

Minor points:

1. “Define the acronyms only once.”
2. “The panels in Fig. 1 are not described in order in the text (e.g., panels c-d are mentioned before panels a-b”
3. “Line 405: 'compare' is repeated twice.
4. “Line 361 ‘dimension d’, d should be in italics.”

We thank the reviewer for pointing out these corrections. The suggested changes are applied to the manuscript.
